# *P*-*S*-*N* Curve Description of Laser Metal Deposition Ti-6.5Al-2Zr-1Mo-1V Titanium Alloy after Duplex Annealing

**DOI:** 10.3390/ma12030418

**Published:** 2019-01-30

**Authors:** Tianshuai Wang, Xiaofan He, Xiaobo Wang, Yuhai Li

**Affiliations:** School of Aeronautic Science and Engineering, Beihang University, Beijing 100083, China; wts@buaa.edu.cn (T.W.); wangxb@buaa.edu.cn (X.W.); lyh601@263.net (Y.L.)

**Keywords:** laser metal deposition, fatigue life distribution, bimodal lognormal distribution, maximum likelihood estimation, *P-S-N* curve

## Abstract

Fatigue tests were conducted on standard smooth laser metal deposition (LMD) Ti-6.5Al-2Zr-1Mo-1V titanium alloy specimens under three constant-amplitude stresses. The mixed failure behaviors and the influence of internal pores on the fatigue life were discussed in detail. The double-peak characteristics of the fatigue life distribution have been observed, and a bimodal lognormal distribution (BLG) with five variables can be used to describe the fatigue life variation. A parameter estimation method based on the maximum likelihood estimation (MLE) method was proposed to estimate the BLG distribution parameters, and the Newton–Raphson algorithm was used to solve the nonlinear equations derived from the likelihood functions. A *P-S-N* curve description of LMD titanium alloy was established based on the BLG and verified by fatigue life data obtained from a fatigue test.

## 1. Introduction

Metal component additive manufacturing (AM) is the general designation for a series of advanced manufacturing processes based on a three-dimensional digital model that are used to fabricate metal components by melting and depositing metal powders or wires layer upon layer. AM can be divided into two main categories according to the different supply methods of the raw metal materials: powder bed fusion (PBF) and direct energy deposition (DED). PBF processes, such as selective laser melting (SLM), use thermal energy to selectively fuse regions of a powder bed, while DED processes use focused thermal energy to fuse materials by melting during deposition [1]. DED methods, including laser metal deposition (LMD), are more suitable for fabricating large titanium structural components than PBF processes [2,3,4,5,6]. Large titanium structural components have been successfully fabricated by many researchers and research institutions by using LMD and have been widely used as load-bearing structures in aeronautical engineering [7,8].

Aeronautical structures experience repeat loads over their long periods of service, and fatigue is one of the main damage phenomena in aerospace metallic structures [9]. Therefore, the fatigue behavior of LMD titanium components must be further elucidated, and fatigue performance characterization methods must be established. The major factors causing the fatigue failure of LMD and other AM titanium alloys include the microstructure, internal defects, and residual stresses [10]. 

The specific thermodynamic process used during manufacturing, i.e., cyclic heating and rapid cooling, results in the unique microstructure of the LMD titanium alloy, which includes relatively large columnar grains, multiple melt layers between the processing layers and unique microstructures [11]. These specific characteristics in the microstructures may have some influence on the fatigue performance of the parts [12,13,14,15]. Wang et al. [12] studied the fatigue strength of LMD TC18(Ti-5Al-5Mo-5V-1Cr-1Fe) titanium alloy and determined that its strength is much lower than that of wrought TC18 titanium alloy. Sterling et al. [13] reported that the fatigue lives of LMD Ti-6Al-4V specimens, including as-built and annealed specimens, are shorter than those of wrought specimens. Zhai et al. [14,15] also determined that LMD Ti-6Al-4V has a lower fatigue crack growth threshold than wrought Ti-6Al-4V. Studies have also been conducted on the fatigue mechanism and the effects of the microstructures of titanium alloys manufactured by LMD and other AM processes. The studies of Rafi et al. [16] and Zhai [13] et al. showed that, compared to EBM Ti-6Al-4V, LMD and SLM Ti-6Al-4V parts have a higher fatigue strength but lower fatigue toughness, and this phenomenon may result from the presence of fine α’ martensite. 

Internal defects are another major factor affecting the fatigue performance. Such defects introduce a stress concentration around them and easily become a nucleation site of crack [17]. Biswas et al. [18] and Li et al. [19] revealed that the internal defects, such as lack of fusion (LOF) and pores, are likely to become nucleation sites for shear bands and micro-cracks. The presence of internal defects may also lead to mixed failure behaviors that were observed in many studies [12,13,17,20,21,22,23,24,25,26], inconsistent with the case of traditionally manufactured titanium alloy. The crack may initiate from both surface and internal defects of specimens. The influences of different types of defects are also investigated. Akerfeldt et al. [20] stated that the internal LOF is more likely to result in cracks than pores, and this phenomenon is particularly significant in specimens with a force directed parallel to the powder deposition direction. Prabhu et al. [21] showed that unmelted particles in LMD titanium alloy specimens have a significant effect on the fatigue life, while the pores have a minimal effect. Leuders et al. [22] considered that the pores within specimens have a drastic effect on the fatigue behavior of SLM titanium alloys in the high-cycle-fatigue (HCF) regime.

The location and size of internal defects also affect the fatigue properties to a great extent. Many investigations have shown that the nearer pores are to the surface, the shorter the fatigue life [13,23,24,25,26]. This phenomenon can be partly explained by Xu et al.’s [27] study. The cross-sectional area of a defect normal to the applied stress has a large effect on the fatigue life properties [28]. Several studies reported that fatigue failure occurs first at the largest defect, so there is a shorter fatigue life for AM specimens in the presence of larger internal defects [13,23,24,25]. However, He et al. [26] indicated a different conclusion that a larger pore may lead to a longer fatigue life when the pores have similar distances to the surface. 

Residual stresses are also a direct cause of crack initiation that can affect the fatigue properties of AM specimens. AM processes, particularly LMD and other laser-based AM processes, are prone to induce significant residual stresses due to their large inherent temperature gradients [29]. Different process parameters and deposition strategies may lead to different residual stress distributions. Tensile residual stresses are always distributed at the surface and near-surface zone of as-built AM titanium alloy parts and may have a significant influence on the fatigue properties [30]. Although the residual stress can be partly relieved by some heat treatment processes, it is hardly eliminated completely [22,31,32,33].

The fatigue behaviors of AM materials are usually complicated due to their unique microstructure, internal defects, and residual stress distributions. As a new manufacturing method, the fatigue life properties must be precisely described. The experimental techniques and description methods for *S-N* curves have long been a concern [34]. The *S-N* curves of materials show the pre-conditions and inputs for anti-fatigue structure design. Fatigue life data always have significant variations, and the fatigue life under a specific stress level is closely related to the survival rate, *P*. In many cases, especially for the reliability design of components, the relationships between the fatigue stress and life at different survival rates, namely, the *P-S-N* curves, must be determined. The previously mentioned *S-N* curve is the median fatigue life curve, i.e., the *P-S-N* curve with a 50% survival rate. Scholars have conducted studies on the fatigue properties of AM materials in recent years, and the *P-S-N* curves (*S-N* curves) of different materials manufactured by several different AM processes have been tested [35,36,37,38,39,40]. Regulations and standards such as ASTM E739-10 [41] and ISO 12107:2012 [42] have been issued with test procedures and method descriptions for *P-S-N* curves (*S-N* curves) based on a lognormal distribution. However, the unique AM fabrication process leads to various failure behaviors, and the fatigue life of AM materials therefore displays significantly greater uncertainty and variation than that of their conventionally manufactured counterparts [26,40,43,44]. The mixed failure behaviors and large variation make the existing description methods unable to accurately describe the fatigue properties of AM materials, and the following problems may occur when the existing description method is used to determine the fatigue *P-S-N* curves of AM materials:

(i) Because of the complex fatigue failure behaviors, the fatigue life distribution of AM materials may be different from that of traditional materials, and the traditional distribution model thus cannot accurately describe the fatigue life variation under specific stress levels.

(ii) Due to the large variation in the fatigue life, the reliability life in the high-reliability region will be very short using the traditional *P-S-N* curve model to describe the fatigue properties of AM materials.

(iii) The large fatigue life variation requires a larger number of fatigue test specimens to determine the *P-S-N* curve, which will inevitably increase the time and economic cost of the process.

In this paper, two sets of fatigue tests under peak stresses of 720 and 760 MPa were conducted, and fatigue life data under three different stress levels were obtained, including the data under 800 MPa published in [26]. Sufficient data, no fewer than 15 specimens under each stress level, were used to develop and prove the conclusions. Compared with the existing research, especially the previous study ([26]), there are four main innovations and contributions in this paper:

(i) This paper examines more data (at least 15 specimens under each stress level) than in previous studies, which generally reported the data of 5–10 specimens under the same stress [23,26,35,37,38,39,40,45]; the data herein were used to illustrate the mixed failure behavior and fatigue life properties.

(ii) A *P-S-N* curve description method of the LMD Ti-6.5Al-2Zr-1Mo-1V titanium alloy was established based on a bimodal lognormal distribution (BLG).

(iii) Considering the disadvantages of the parameter estimation method based on rank distribution theory developed in [26], especially the weakness in robustness, the maximum likelihood estimation (MLE) method was used to estimate the parameters, and the Newton–Raphson algorithm was used to solve the equations.

(iv) Compared to previous studies [13,23,24,25,26], the mixed failure behavior and the influence of internal pores on the fatigue life of LMD Ti-6.5Al-2Zr-1Mo-1V specimens were discussed in more detail, and the conclusion regarding the influence of the size and location of the pore defects on the fatigue life was corrected by analyzing the greater amount of data in this paper.

## 2. Materials and Experiments

### 2.1. Specimens

**Figure 1:**
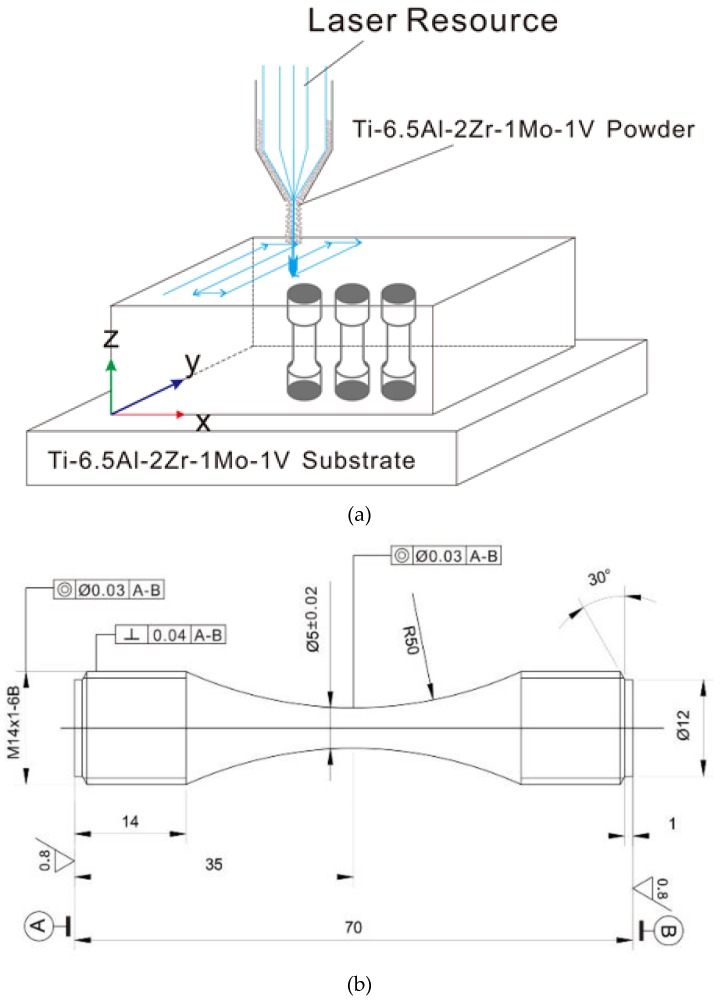
(**a**) Schematic drawing of Ti-6.5Al-2Zr-1Mo-1V blank fabrication by Laser Metal Deposition (LMD) and cylindrical specimen extraction. (**b**) Schematic illustration of the specimen dimensions in mm [26].

The Ti-6.5Al-2Zr-1Mo-1V titanium alloy specimens used in this paper were fabricated in the same batch as those in [26] with the same manufacturing procedures. A Ti-6.5Al-2Zr-1Mo-1V titanium alloy rectangular plate was deposited on a substrate made of wrought Ti-6.5Al-2Zr-1Mo-1V titanium alloy, as shown in Figure 1a. The spherical Ti-6.5Al-2Zr-1Mo-1V powder used for the LMD was prepared by a plasma rotation electrode process. The Ti-6.5Al-2Zr-1Mo-1V titanium alloy plate was machined into standard smooth cylindrical specimens after duplex annealing (850 °C AC 1 h, 650 °C FC 2 h), as shown in Figure 1b, and had a surface roughness, *Ra*, of 0.32 μm. 

**Figure 2:**
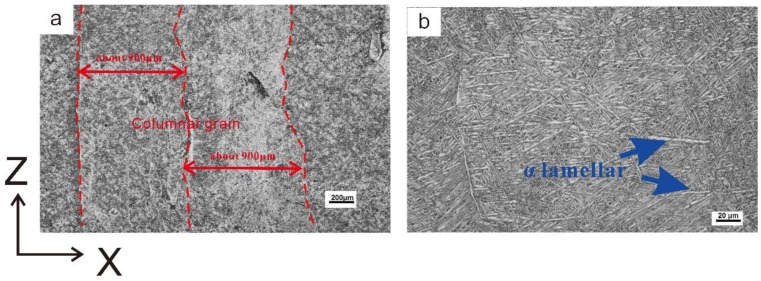
Microstructure of laser metal deposition (LMD) Ti-6.5Al-2Zr-1Mo-1V, (**a**):50X; (**b**):500X.

Figure 2 shows the microstructure of the material. The material mainly consists of coarse columnar grains with the growth direction in the Z direction. Figure 2a shows that the size of the columnar grains is approximately 900 μm. As shown in Figure 2b, the columnar grains mainly consist of a basket-weave microstructure, and a large number of *α* colonies are present near the grain boundary. However, Figure 2b also shows that the microstructure of the material is inhomogeneous. In addition to the fine basket-weave organization, an *α* lamellar microstructure also exists in the material. 

### 2.2. Experiments

The fatigue test was performed on an Instron 8801-100kN electrohydraulic servo fatigue system in an atmospheric environment at room temperature under a sine-wave constant-amplitude (CA) stress with a stress ratio of 0.06 and frequency of 10 Hz, the same experimental procedures and conditions used in [26]. Two sets of fatigue tests with peak stresses of 720 and 760 MPa were carried out. Therefore, three sets of fatigue test data were obtained in total, including the data under 800 MPa in [26]. Fracture surface examinations and fractographic analyses were performed by scanning electron microscopy (SEM, JEOL, Tokyo, Japan/Carl Zeiss, Jena, Germany) after the fatigue tests, and the crack initiation and failure forms were identified. 

## 3. Tests Results

Two sets of fatigue tests were conducted under two different stress levels and combined with the test results in [26]. Fifteen, 17, and 22 specimens under 720, 760, and 800 MPa, respectively, were analyzed in total. Fracture surface examinations and fractographic analyses were performed, and mixed failure behaviors appeared in all three sets of fatigue tests. All the fracture surfaces were classified into two categories according to the crack initiation locations, and the fatigue life distribution was preliminarily estimated based on a lognormal distribution.

### 3.1. Fractographic Analysis

**Figure 3:**
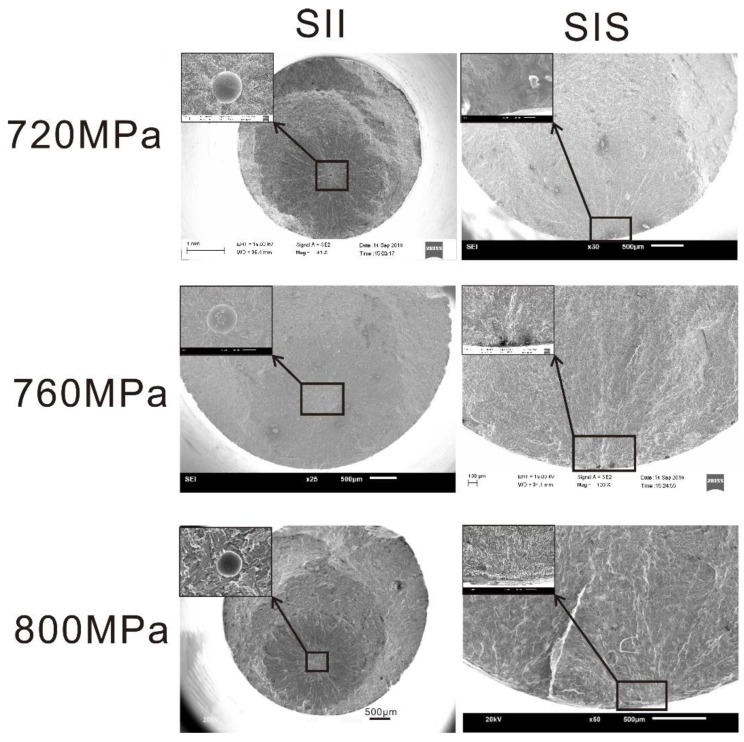
Typical fracture surfaces of specimens with internal crack initiation (SII) and specimens with crack initiating from suface (SIS) under three stress levels.

The fatigue lives of all specimens were in the range of the medium- and high-cycle fatigue regimes. The fractographic, in Figure 3, shows that mixed failure behaviors appeared under the three stress levels. Parts of the specimens, with internal crack initiation, fractured due to cracks initiating from internal defects such as internal pores were denoted as SII. The other parts of the specimens that fractured due to cracks initiating from the surface or subsurface of the specimens were denoted as SIS. 

**Figure 4:**
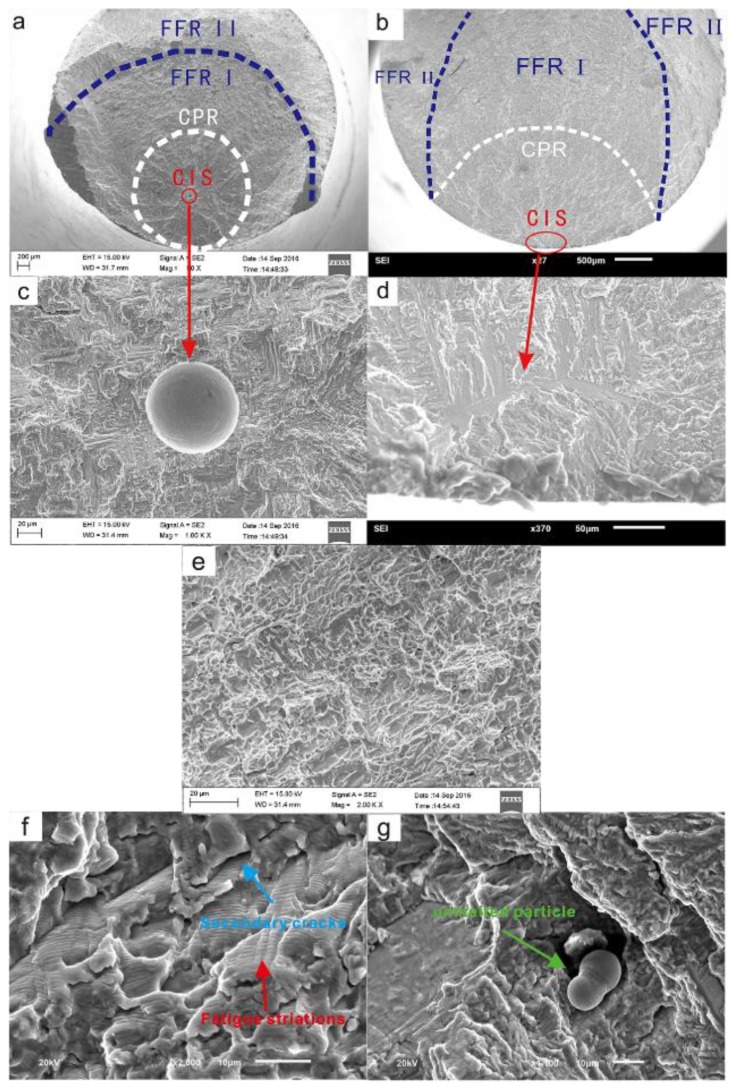
Typical fracture photographs of (**a**) SII and (**b**) SIS and fracture surface region details of (**c**) SII and (**d**) SIS; (**e**) typical dimple feature in the fast fracture region (FFR); (**f**) typical fatigue striations and (**g**) secondary cracks and unmelted particles in the crack propagation region (CPR).

For SII, the crack origins were all pores, and no LOF was observed in this investigation. A typical fracture photograph of SII is shown in Figure 4a,c. The center of the region close to the initiation of the crack is bright, while the surrounding area is dark. For SIS, the crack origins are usually mechanical scratches and *α* lamellar structures located on the surface or subsurface of the specimens. A typical fracture photograph of SIS is shown in Figure 4b,d. Significant radial ridges can be seen on the fracture surface, extending radially from the origin site to the surrounding area.

The fracture surfaces of SII and SIS can be divided into three regions, as shown in Figure 4a,b:

(i) crack initiation site (CIS): the crack initiation location; 

(ii) crack propagation region (CPR): a flat plane vertical to the load direction that forms as the result of the crack-stabilized propagation;

(iii) fast fracture region (FFR): the area produced by fast crack propagation and fracture.

CIS and CPR are relatively flat and nearly in the same plane. As shown in Figure 4c,d, the FFR can be divided into two parts, denoted as FFR I and FFR II. FFR I is relatively flat and roughly in the same plane as CIS and CPR, while FFR II is a plane inclined at an angle of approximately 45° relative to the plane of the CIS and CPR. Different stress–strain states when fracture occurs may lead to the differences in the morphology of the two parts of the FFR. FFR I is close to the ideal plane strain state due to constraints inside the materials, while FFR II is similar to the plane stress state because of the small distance from the surface of the specimen. Obvious dimple features can be seen in the FFR (Figure 4e).

The characteristics of the CPR are almost the same for SIS and SII. The typical characteristics are shown in Figure 4e,f. Fatigue striations and secondary cracks were found in the CPR (Figure 4e). Unmelted particles were observed, and their location and shape were irregular (Figure 4f). The formation of secondary cracks may be related to the laser metal deposition (LMD) parameters, heat treatment process, and stress state. Some powder was not completely melted and remained as unmelted particles, which may have a spherical shape or be present as partially melted irregular particles. These particles produced irregular voids inside the specimen, and these voids could affect the fatigue properties of the specimen.

**Figure 5:**
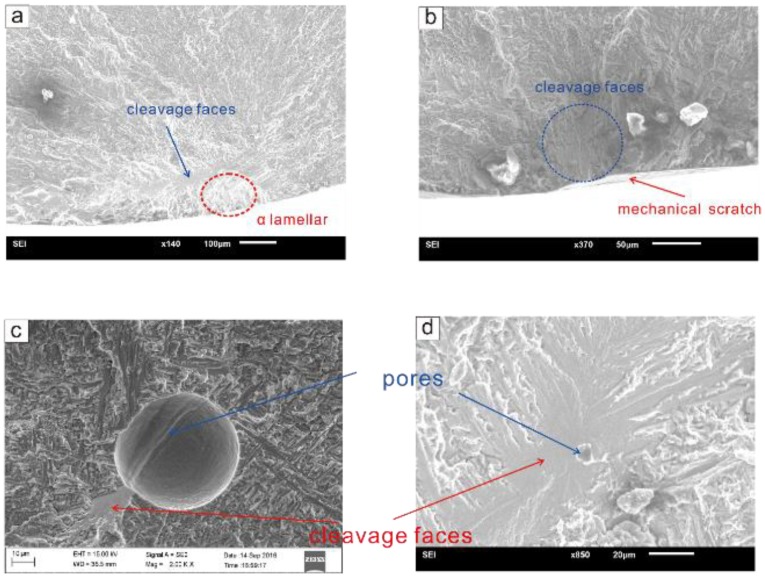
Details of CIS of (**a**,**b**) SIS; (**c**,**d**) SII.

For SIS, cracks initiate from coarse α lamellar grains or mechanical scratches located on the surface or subsurface of the specimens, as shown in Figure 5a,b. Mechanical scratches may be traces left by tools during the machining process. Figure 5a,b show apparent cleavage faces near the CIS, and cleavage feather-like features can be easily identified. 

For SII specimens, as shown in Figure 5, cracks originate from internal pores. The sizes of the pores inducing the cracks vary greatly between different specimens. Figure 5c,d are typical fracture photographs of two SII specimens. In Figure 5c, the pore area is relatively large, and many discontinuous cleavage faces can be seen near the pore. In Figure 5d, the pore area is small, and a continuous cleavage face can be seen around the pore. Thus, the crack initiation mechanism induced by the different pore sizes may be different. 

### 3.2. Fatigue Life Data

**Figure 6:**
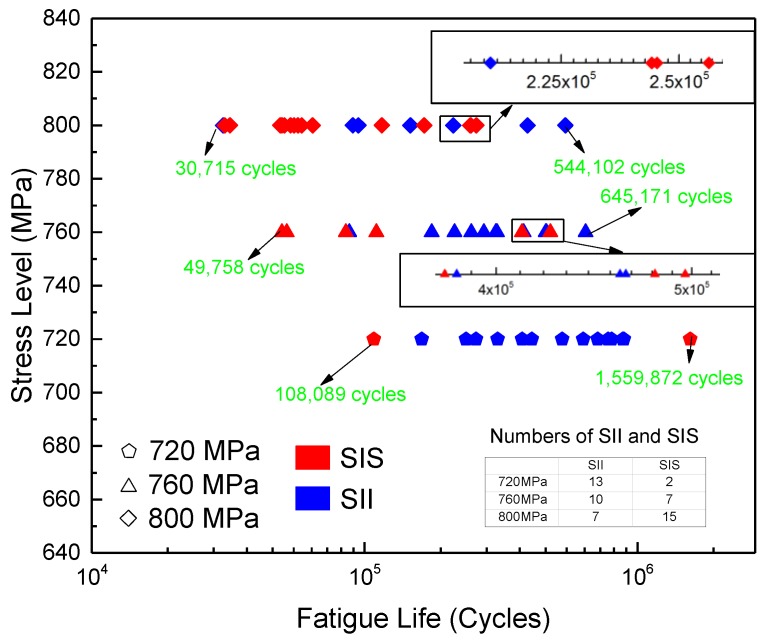
Fatigue life data under three stress levels.

The fatigue life data under three stress levels obtained from the fatigue tests are shown in Figure 6. The pentagons represent the fatigue lives of the specimens under 720 MPa, while the triangles and diamonds represent those of the specimens under 760 and 800 MPa, respectively. The fatigue lives of all specimens are in the range of medium and long fatigue lifetimes. The ranges of the fatigue lives are 108,089–1,559,872 cycles (720 MPa), 49,758–645,171 cycles (760 MPa), and 30,715–544,102 cycles (800 MPa), respectively. The data dots representing the fatigue life of SII are blue, while those of SIS are red. The fatigue lives of SII and SIS overlap with each other. Therefore, the fatigue lives of SII and SIS cannot be differentiated using the location and type of CIS. 

The fatigue life is denoted as the random variable *Y*. Conventionally, a lognormal distribution (LG) can be used to describe the fatigue life distribution of metal materials. Assuming that the fatigue life follows an LG, the probability density function (PDF), $f_{Y}\left(y\right)$, and the cumulative distribution function (CDF), $F_{Y}\left(y\right)$, of *Y* are given by Equations (1) and (2), respectively.

(1) $$f_{Y}\left(y\right)=\left\{ \begin{array}{ll} \frac{1}{\sqrt{2\pi }\cdot \sigma_{\mathrm{LG}}\cdot y\cdot \ln10}\exp\left[-\frac{1}{2}{\left(\frac{\mathrm{lg}y-\mu_{\mathrm{LG}}}{\sigma_{\mathrm{LG}}}\right)}^{2}\right] & y>0,\\ 0 & y\leq 0. \end{array}\right.$$

(2) $$F_{Y}\left(y\right)=\left\{ \begin{array}{ll} \frac{1}{\sqrt{2\pi }\cdot \sigma_{\mathrm{LG}}\cdot \ln10}\int_{0}^{y}\frac{1}{y}\exp\left[-\frac{1}{2}{\left(\frac{\mathrm{lg}y-\mu_{\mathrm{LG}}}{\sigma_{\mathrm{LG}}}\right)}^{2}\right]dy & y>0,\\ 0 & y\leq 0. \end{array}\right.$$

(3) $$\left\{ \begin{array}{l} \mu_{\mathrm{LG}}=\frac{1}{n}\sum_{i=1}^{n}\mathrm{lg}y_{i},\\ \sigma_{\mathrm{LG}}=\sqrt{\frac{1}{n-1}\sum_{i=1}^{n}{\left(\mathrm{lg}y_{i}-\mu_{\mathrm{LG}}\right)}^{2}},\\ N_{50,\mathrm{LG}}=10^{\mu_{\mathrm{LG}}}. \end{array}\right.$$

Two parameters, the logarithmic expectation (*μ*_LG_) and logarithmic life standard deviation (*σ*_LG_), can be used to determine the distribution. The parameters under the three stress levels were estimated by using the MLE method by using Equation (3) and are listed in Table 1. The table also contains the maximum and minimum values of the fatigue life and the logarithmic median life, $N_{50,\mathrm{LG}}$ under the three stress levels. Table 1 shows that the *σ*_LG_ values under all three stress levels are no less than 0.3 and increase with the stress level, indicating that the fatigue life has a large variation. The maximum fatigue life under each stress level is approximately 15 times the minimum value. The variation may be caused by the mixed failure behavior described in Section 3.1.

**Figure 7:**
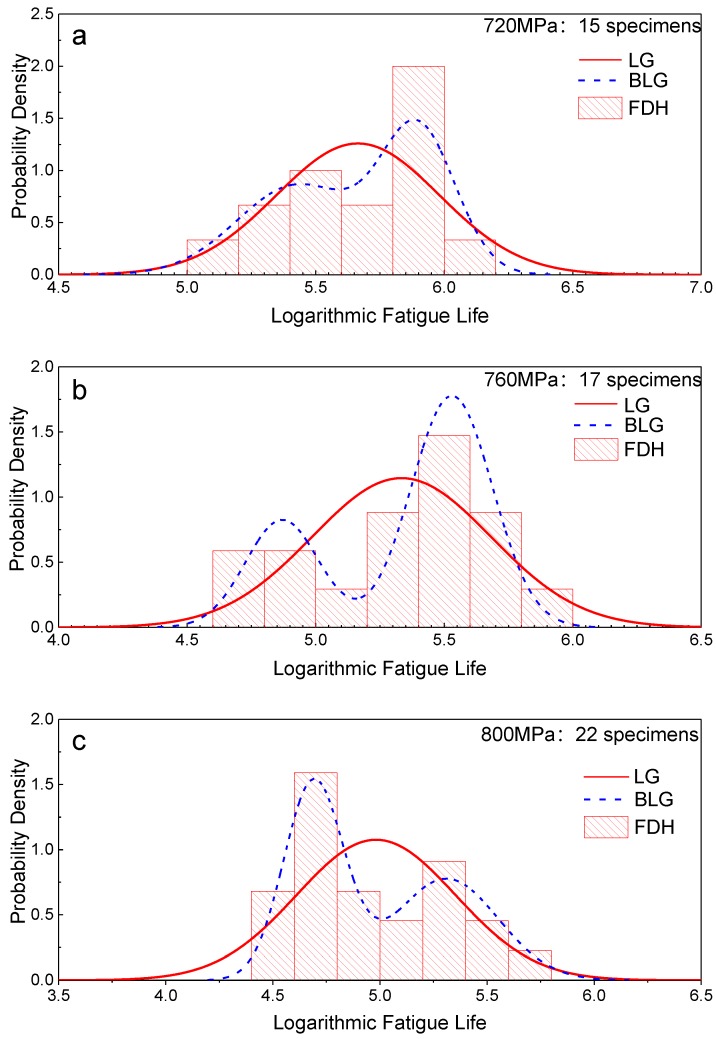
Probability density function (PDF) curves and frequency distribution histograms (FDHs) of specimens under (**a**) 720, (**b**) 760, and (**c**) 800 MPa [26].

To further elucidate the distribution of the fatigue life, frequency distribution histograms (FDHs) of the logarithmic lives under the three stress levels are plotted in Figure 7 with an interval of 0.2. The estimated PDF curves are also plotted in the figure (red curves). The PDF curves of the LG have significant differences from the FDHs under the three stress levels, and the FDHs have apparent double-peak characteristics. Therefore, the fatigue life distribution of LMD titanium alloy cannot be well fitted by using an LG. 

## 4. Bimodal Lognormal Distribution

Since the PDFs of the LG in Figure 7 cannot fit the FDHs of the fatigue life data of the specimens, the LG distribution is not suitable to describe the fatigue life of the LMD titanium alloy and will lead to large fatigue life variations. The poor description result of the LG and the significant variation induced by the unsuitable distribution may lead to very short reliability lives under high-reliability demands and may increase the requirement of the number of specimens in the fatigue tests. Thus, a distribution model that can describe the fatigue life of LMD titanium alloy more precisely is needed.

In this section, a bimodal lognormal distribution (BLG), proposed in [26], is used to describe the fatigue life data of LMD titanium alloys. Considering the disadvantages of the parameter estimation method developed in [26], the Newton–Raphson algorithm is used to estimate the parameters based on the MLE method.

### 4.1. Distribution Model

Assume that the fatigue life, *Y*, follows a BLG, $Y∼BLG(\alpha ,\mu_{1},\sigma_{1}^{2},\mu_{2},\sigma_{2}^{2})$, where $\alpha ,\mu_{1},\sigma_{1},\mu_{2},\sigma_{2}$ are the five distribution parameters of the BLG. The PDF, $f_{Y}\left(y\right)$, and the CDF, $F_{Y}\left(y\right)$, of *Y* are given by Equation (4), where *α* represents the weight and 0≤α≤1.

(4) $$\left\{ \begin{array}{l} f_{Y}\left(y\right)=\alpha f_{Y_{1}}\left(y\right)+(1-\alpha )f_{Y_{2}}\left(y\right),\\ F_{Y}\left(y\right)=\alpha F_{Y_{1}}\left(y\right)+(1-\alpha )F_{Y_{2}}\left(y\right). \end{array}\right.$$

*Y*_1_ and *Y*_2_ are two independent random variables that follow a lognormal distribution $\left\{ \begin{array}{l} Y_{1}∼LG(\mu_{1},\sigma_{1}^{2})\\ Y_{2}∼LG(\mu_{2},\sigma_{2}^{2}) \end{array}\right.$. The PDF and CDF of *Y*_1_ and *Y*_2_ are shown in Equations (5) and (6), respectively.

(5) $$f_{Y_{i}}\left(y\right)=\left\{ \begin{array}{ll} \frac{1}{\sqrt{2\pi }\cdot \sigma_{i}\cdot y\cdot \ln10}\exp\left[-\frac{1}{2}{\left(\frac{\mathrm{lg}y-\mu_{i}}{\sigma_{i}}\right)}^{2}\right] & y>0\\ 0 & y\leq 0 \end{array}\right.(i=1,2),$$

(6) $$F_{Y_{i}}\left(y\right)=\left\{ \begin{array}{ll} \frac{1}{\sqrt{2\pi }\cdot \sigma_{i}\cdot \ln10}\int_{0}^{y}\frac{1}{y}\exp\left[-\frac{1}{2}{\left(\frac{\mathrm{lg}y-\mu_{i}}{\sigma_{i}}\right)}^{2}\right]dy & y>0\\ 0 & y\leq 0 \end{array}\right.(i=1,2).$$

### 4.2. BLG Parameter Estimation Method

In [26], a parameter estimation method based on the rank statistics theory is proposed. The basic principle of this method is that the minimization of the Sum of Squared Error (SSE) is used as a criterion for determining the distribution parameters. This method is easy to perform but lacks the rigor of mathematical logic. It also has some weakness in robustness and can easily be affected by the sample alteration. A relatively large sample size is needed. Considering the above shortcomings of the method, the MLE method was used to estimate the parameters. The likelihood function is constructed, and the Newton–Raphson method is used to obtain an estimated value for each parameter. The following are the detailed steps.

For fatigue life data under a specific stress level, the MLE method can be used to estimate the five distribution parameters $\alpha ,\mu_{1},\sigma_{1},\mu_{2},\sigma_{2}$. To simplify the expression, set *X* = lg*Y*. The random variable *X* follows a bimodal normal distribution (BNG), denoted as $X∼BNG(\alpha ,\mu_{1},\sigma_{1}^{2},\mu_{2},\sigma_{2}^{2})$. The PDF and CDF are given by Equation (7), and the PDF and CDF of *Y*_1_ and *Y*_2_ are determined by Equations (8) and (9), respectively. Obviously, the BLG has the same parameters as the BNG.

(7) $$\left\{ \begin{array}{l} f_{X}\left(x;\alpha ,\mu_{1},\sigma_{1},\mu_{2},\sigma_{2}\right)=\alpha f_{X_{1}}\left(x;\mu_{1},\sigma_{1}\right)+(1-\alpha )f_{X_{2}}\left(x;\mu_{2},\sigma_{2}\right),\\ F_{X}\left(x;\alpha ,\mu_{1},\sigma_{1},\mu_{2},\sigma_{2}\right)=\alpha F_{X_{1}}\left(x;\mu_{1},\sigma_{1}\right)+(1-\alpha )F_{X_{2}}\left(x;\mu_{2},\sigma_{2}\right), \end{array}\right.$$

(8) $$f_{X_{i}}\left(x;\mu_{i},\sigma_{i}\right)=\left\{ \begin{array}{ll} \frac{1}{\sqrt{2\pi }\cdot \sigma_{i}\cdot x}\exp\left[-\frac{1}{2}{\left(\frac{x-\mu_{i}}{\sigma_{i}}\right)}^{2}\right] & x>0\\ 0 & x\leq 0 \end{array}\right.(i=1,2),$$

(9) $$F_{X_{i}}\left(x;\mu_{i},\sigma_{i}\right)=\left\{ \begin{array}{ll} \frac{1}{\sqrt{2\pi }\cdot \sigma_{i}}\int_{0}^{x}\frac{1}{x}\exp\left[-\frac{1}{2}{\left(\frac{x-\mu_{i}}{\sigma_{i}}\right)}^{2}\right]dx & x>0\\ 0 & x\leq 0 \end{array}\right.(i=1,2).$$

The likelihood function, $L(x;\alpha ,\mu_{1},\sigma_{1},\mu_{2},\sigma_{2})$, can be expressed as Equation (10),

(10) $$L(x;\alpha ,\mu_{1},\sigma_{1},\mu_{2},\sigma_{2})=\prod_{i=1}^{n}\left[\alpha f_{X}(x;\mu_{1},\sigma_{1})+(1-\alpha )f_{X}(x;\mu_{2},\sigma_{2})\right].$$

By taking the logarithm of both sides of Equation (10), the logarithmic likelihood function can be obtained as Equation (11),

(11) $$\ln L(x;\alpha ,\mu_{1},\sigma_{1},\mu_{2},\sigma_{2})=\sum_{i=1}^{n}\ln\left[\alpha f_{X}(x_{i};\mu_{1},\sigma_{1})+(1-\alpha )f_{X}(x_{i};\mu_{2},\sigma_{2})\right].$$

The first partial derivative of $\ln L(x;\alpha ,\mu_{1},\sigma_{1},\mu_{2},\sigma_{2})$ can then be taken with respect to $\alpha ,\mu_{1},\sigma_{1},\mu_{2},\sigma_{2}$, respectively. If the partial derivatives are equal to zero, then

(12) $$\left\{ \begin{array}{c} \frac{\partial \ln L}{\partial \alpha }=\sum_{i=1}^{n}\frac{f_{X}(x;\mu_{1},\sigma_{1})-f_{X}(x;\mu_{2},\sigma_{2})}{\alpha f_{X}(x_{i};\mu_{1},\sigma_{1})+(1-\alpha )f_{X}(x_{i};\mu_{2},\sigma_{2})}=0,\\ \frac{\partial \ln L}{\partial \mu_{1}}=\sum_{i=1}^{n}\frac{\alpha }{\alpha f_{X}(x_{i};\mu_{1},\sigma_{1})+(1-\alpha )f_{X}(x_{i};\mu_{2},\sigma_{2})}•\frac{\left(x_{i}-\mu_{1}\right)}{\sqrt{2\pi }\sigma_{1}^{3}}\exp\left[-\frac{{\left(x_{i}-\mu_{1}\right)}^{2}}{2\sigma_{1}^{2}}\right]=0,\\ \frac{\partial \ln L}{\partial \sigma_{1}}=\sum_{i=1}^{n}\frac{\alpha }{\alpha f_{X}(x_{i};\mu_{1},\sigma_{1})+(1-\alpha )f_{X}(x_{i};\mu_{2},\sigma_{2})}•\left\{-\frac{1}{\sqrt{2\pi }\sigma_{1}^{2}}\exp\left[-\frac{{\left(x_{i}-\mu_{1}\right)}^{2}}{2\sigma_{1}^{2}}\right]+\frac{{\left(x_{i}-\mu_{1}\right)}^{2}}{\sqrt{2\pi }\sigma_{1}^{4}}\exp\left[-\frac{{\left(x_{i}-\mu_{1}\right)}^{2}}{2\sigma_{1}^{2}}\right]\right\}=0,\\ \frac{\partial \ln L}{\partial \mu_{2}}=\sum_{i=1}^{n}\frac{1-\alpha }{\alpha f_{X}(x_{i};\mu_{1},\sigma_{1})+(1-\alpha )f_{X}(x_{i};\mu_{2},\sigma_{2})}•\frac{\left(x_{i}-\mu_{2}\right)}{\sqrt{2\pi }\sigma_{2}^{3}}\exp\left[-\frac{{\left(x_{i}-\mu_{2}\right)}^{2}}{2\sigma_{2}^{2}}\right]=0,\\ \frac{\partial \ln L}{\partial \sigma_{2}}=\sum_{i=1}^{n}\frac{1-\alpha }{\alpha f_{X}(x_{i};\mu_{1},\sigma_{1})+(1-\alpha )f_{X}(x_{i};\mu_{2},\sigma_{2})}•\left\{-\frac{1}{\sqrt{2\pi }\sigma_{2}^{2}}\exp\left[-\frac{{\left(x_{i}-\mu_{2}\right)}^{2}}{2\sigma_{2}^{2}}\right]+\frac{{\left(x_{i}-\mu_{2}\right)}^{2}}{\sqrt{2\pi }\sigma_{2}^{4}}\exp\left[-\frac{{\left(x_{i}-\mu_{2}\right)}^{2}}{2\sigma_{2}^{2}}\right]\right\}=0 \end{array}\right.$$

The equations in Equation (12) can be simplified and denoted as $f_{L,i}(x;\alpha ,\mu_{1},\sigma_{1},\mu_{2},\sigma_{2}),i=1,2,\cdots ,5$:

(13) $$\left\{ \begin{array}{c} f_{L,1}(x;\alpha ,\mu_{1},\sigma_{1},\mu_{2},\sigma_{2})=\sum_{i=1}^{n}\frac{f_{X}(x;\mu_{1},\sigma_{1})-f_{X}(x;\mu_{2},\sigma_{2})}{\alpha f_{X}(x_{i};\mu_{1},\sigma_{1})+(1-\alpha )f_{X}(x_{i};\mu_{2},\sigma_{2})}=0\\ f_{L,2}(x;\alpha ,\mu_{1},\sigma_{1},\mu_{2},\sigma_{2})=\sum_{i=1}^{n}\frac{\alpha f_{X}(x_{i};\mu_{1},\sigma_{1})}{\alpha f_{X}(x_{i};\mu_{1},\sigma_{1})+(1-\alpha )f_{X}(x_{i};\mu_{2},\sigma_{2})}•\frac{\left(x_{i}-\mu_{1}\right)}{\sigma_{1}^{2}}=0\\ f_{L,3}(x;\alpha ,\mu_{1},\sigma_{1},\mu_{2},\sigma_{2})=\sum_{i=1}^{n}\frac{\alpha f_{X}(x_{i};\mu_{1},\sigma_{1})}{\alpha f_{X}(x_{i};\mu_{1},\sigma_{1})+(1-\alpha )f_{X}(x_{i};\mu_{2},\sigma_{2})}•\frac{1}{\sigma_{1}}•\left[\frac{{\left(x_{i}-\mu_{1}\right)}^{2}}{\sigma_{1}^{3}}-1\right]=0\\ f_{L,4}(x;\alpha ,\mu_{1},\sigma_{1},\mu_{2},\sigma_{2})=\sum_{i=1}^{n}\frac{\left(1-\alpha )f_{X}(x_{i};\mu_{2},\sigma_{2}\right)}{\alpha f_{X}(x_{i};\mu_{1},\sigma_{1})+(1-\alpha )f_{X}(x_{i};\mu_{2},\sigma_{2})}•\frac{\left(x_{i}-\mu_{2}\right)}{\sigma_{2}^{2}}=0\\ f_{L,5}(x;\alpha ,\mu_{1},\sigma_{1},\mu_{2},\sigma_{2})=\sum_{i=1}^{n}\frac{\left(1-\alpha )f_{X}(x_{i};\mu_{2},\sigma_{2}\right)}{\alpha f_{X}(x_{i};\mu_{1},\sigma_{1})+(1-\alpha )f_{X}(x_{i};\mu_{2},\sigma_{2})}•\frac{1}{\sigma_{2}}•\left[\frac{{\left(x_{i}-\mu_{2}\right)}^{2}}{\sigma_{2}^{3}}-1\right]=0 \end{array}.\right.$$

Equation (13) is thus a nonlinear system with five variables, and obtaining an analytical solution is difficult. Thus, the Newton–Raphson iterative algorithm was used to solve this nonlinear system. Let $F_{L}(\theta )={\left(f_{L,1}(\theta ),f_{L,2}(\theta ),f_{L,3}(\theta ),f_{L,4}(\theta ),f_{L,5}(\theta )\right)}^{T}=0$ and $\theta ={\left(\alpha ,\mu_{1},\sigma_{1},\mu_{2},\sigma_{2}\right)}^{T}$. Assuming that the system has been iterated k times, $\theta^{\left(k+1\right)}={\left(\alpha^{\left(k+1\right)},\mu_{1}{}^{\left(k+1\right)},\sigma_{1}{}^{\left(k+1\right)},\mu_{2}{}^{\left(k+1\right)},\sigma_{2}{}^{\left(k+1\right)}\right)}^{T}$ can be calculated by Equation (14).

(14) $$\theta^{\left(k+1\right)}=\theta^{\left(k\right)}-J^{-1}F_{L}(\theta^{\left(k\right)}),k=0,1,2\cdots$$

where **J** represents the derivative of $F_{T}(\theta )$ at $\theta^{\left(k\right)}$(Equation (15)).

(15) $$J=[\begin{array}{c} \frac{\partial f_{L,1}}{\partial \alpha },\frac{\partial f_{L,1}}{\partial \mu_{1}},\frac{\partial f_{L,1}}{\partial \sigma_{1}},\frac{\partial f_{L,1}}{\partial \mu_{2}},\frac{\partial f_{L,1}}{\partial \sigma_{2}}\\ \frac{\partial f_{L,2}}{\partial \alpha },\frac{\partial f_{L,2}}{\partial \mu_{1}},\frac{\partial f_{L,2}}{\partial \sigma_{1}},\frac{\partial f_{L,2}}{\partial \mu_{2}},\frac{\partial f_{L,2}}{\partial \sigma_{2}}\\ \frac{\partial f_{L,3}}{\partial \alpha },\frac{\partial f_{L,3}}{\partial \mu_{1}},\frac{\partial f_{L,3}}{\partial \sigma_{1}},\frac{\partial f_{L,3}}{\partial \mu_{2}},\frac{\partial f_{L,3}}{\partial \sigma_{2}}\\ \frac{\partial f_{L,4}}{\partial \alpha },\frac{\partial f_{L,4}}{\partial \mu_{1}},\frac{\partial f_{L,4}}{\partial \sigma_{1}},\frac{\partial f_{L,4}}{\partial \mu_{2}},\frac{\partial f_{L,4}}{\partial \sigma_{2}}\\ \frac{\partial f_{L,5}}{\partial \alpha },\frac{\partial f_{L,5}}{\partial \mu_{1}},\frac{\partial f_{L,5}}{\partial \sigma_{1}},\frac{\partial f_{L,5}}{\partial \mu_{2}},\frac{\partial f_{L,5}}{\partial \sigma_{2}} \end{array}].$$

In this calculation procedure, the iterative formula in Equation (14) is needed to calculate the inverse matrix of **J**, resulting in a large calculation. Thus, Equation (16) was used in the actual calculation.

(16) $$\left\{ \begin{array}{l} J(\theta^{\left(k\right)})\Delta \theta^{\left(k\right)}=-F_{L}(\theta^{\left(k\right)})\\ \theta^{\left(k+1\right)}=\theta^{\left(k\right)}+\Delta \theta^{\left(k\right)} \end{array}\right..$$

### 4.3. BLG Parameter Estimation Method

Using the MLE method in Section 4.2, the parameters of the BLG distribution were estimated for the fatigue life data obtained from the fatigue tests under three stress levels. The estimated parameters are shown in Table 2. The PDF curves are shown in Figure 7 (blue curves), and the figure shows that the BLG can more effectively and accurately reflect the double-peak characteristics of the fatigue life distribution of LMD titanium alloys. Table 2 shows that *μ*_1_ and *μ*_2_ both decrease with an increase in the stress level. However, *σ*_1_ and *σ*_2_ show different behaviors: the *σ*_1_ values decrease while the *σ*_2_ values increase with an increase in the stress level. 

## 5. *P*-*S*-*N* Curve of LMD Titanium Alloy

A BLG can reflect the fatigue life distribution of LMD titanium alloys more realistically than an LG, so the *P-S-N* curve description method was established based on a BLG model. In the calculation of the *P-S-N* curve, the fatigue life under a specific reliability should be calculated first. Due to cost and time constraints, about only 20 specimens were tested under each stress level. Therefore, the confidence level of the fatigue life under a specific reliability should be considered. 

### 5.1. Fatigue Life Under a Specific Reliability Value

For a specific reliability value, *P*, the fatigue life is denoted as $N_{P}$ and satisfies Equation (17).

(17) $$P(y\geq N_{P})=P,$$

so

(18) $$1-P=\alpha F_{Y_{1}}\left(N_{P}\right)+(1-\alpha )F_{Y_{2}}\left(N_{P}\right).$$

**Figure 8:**
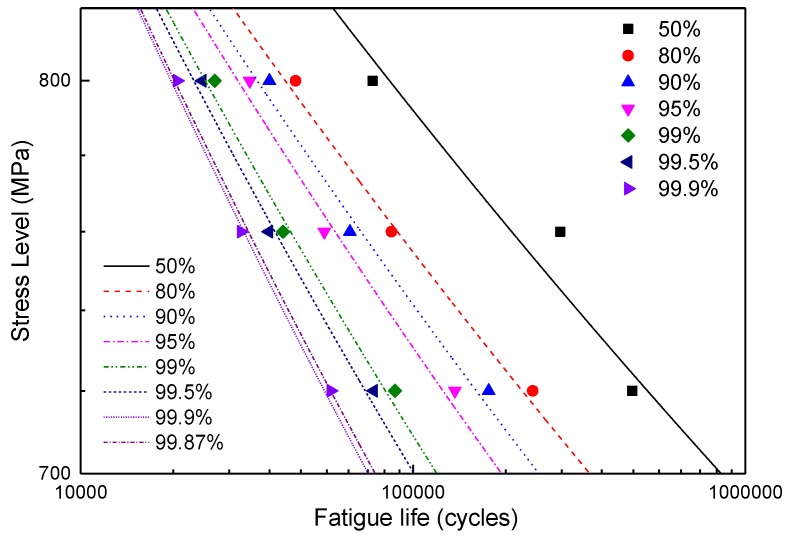
Fatigue lives under typical reliabilities based on a BLG.

Several $N_{P}$ values under the three stress levels were calculated by using a numerical method according to Equation (18) and plotted as double logarithmic coordinates in Figure 8.

### 5.2. P-S-N Curves

Figure 8 shows that the reliability fatigue lives, $N_{P}$, of three stress levels at the same reliability value, *P*, are approximately linear. Thus, the *P-S-N* curve of LMD titanium alloy can be described by the Basquin equation, Equation (19), where S represents the peak stress, and *C_P_* and *m_P_* are two undetermined parameters. Particularly, when *P* = 50%, Equation (19) simplifies to an *S-N* curve.

(19) $$S^{m_{P}}N_{P}=C_{P},$$

and, by taking the logarithm of both sides of Equation (19), the resulting Equation (20) is a linear function. For the specific reliability value *P*, the undetermined parameters *C_P_* and *m_P_* in Equation (20) can be estimated using the linear regression method.

(20) $$m_{P}\mathrm{lg}S+\mathrm{lg}N_{P}=\mathrm{lg}C_{P}.$$

Using the fatigue life data obtained in this paper, the parameters of the *P-S-N* curve under several typical reliabilities were estimated based on the above method and are listed in Table 3 with their correlation coefficients, *r*. Table 3 shows that both parameters, *C_P_* and *m_P_*, decrease with an increase in the reliability value, *P*. The *P-S-N* curves are plotted in Figure 8.

## 6. Discussion

### 6.1. Mixed Failure Behaviors and BLG

The previous studies show that the fatigue behaviors of LMD titanium alloys are different from those of traditional materials, and mixed failure behaviors appear [20,21,22,26,36,46,47]. The investigations in this paper confirmed this view and obtained a more general conclusion. Through the analysis of an abundance of data, the relationship between the stress level and the proportion of SII in the overall population, which has rarely been examined, was obtained herein.

As shown in Figure 3, mixed failure behaviors appeared under the three stress levels. Thus, in the medium- and high-cycle fatigue regimes, it may be an intrinsic characteristic of LMD titanium alloys under CA stress and independent of the stress level. However, the proportion of SII in the overall population may be related to the stress level. In Table 4, *n*_SIS_ and *n*_SII_ represent the numbers of SIS and SII, respectively. The value of *n*_SII_/*n* decreases with an increase in the stress level in an approximately linearly manner. This phenomenon may be caused by the combined influences of the microstructure, the types and geometric parameters of the defects, and the local stress state, although further research is needed on the interpretation of this phenomenon.

**Figure 9:**
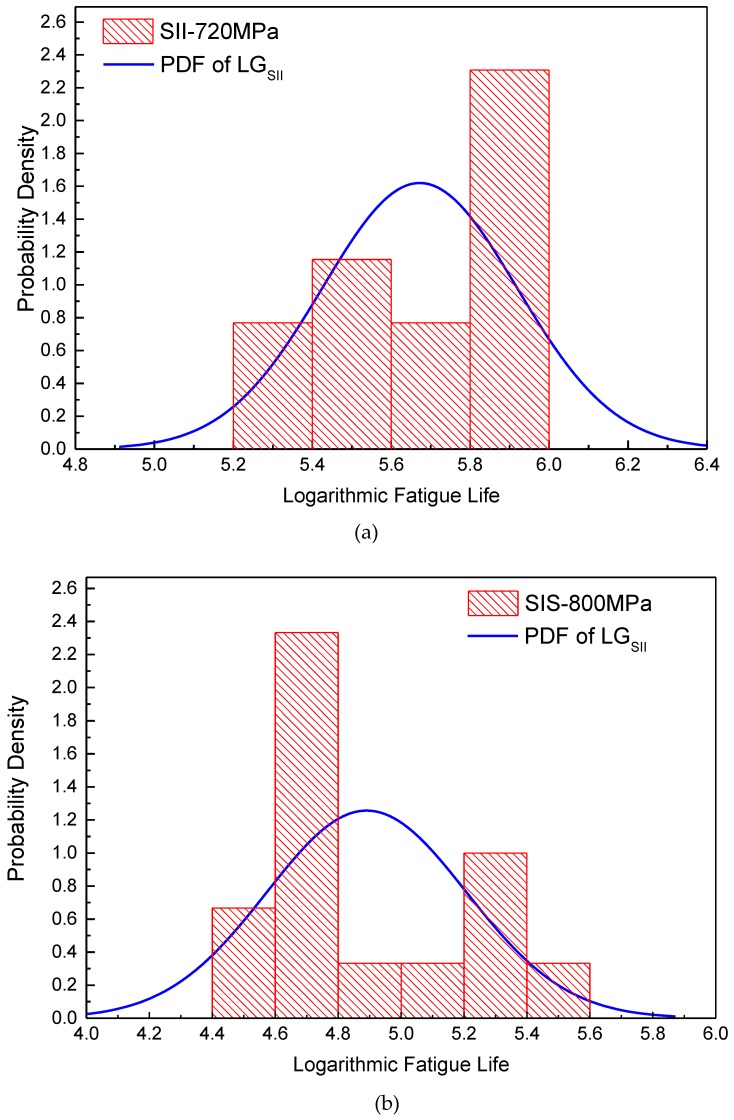
(**a**) FDH of SII under 720 MPa and its PDF; (**b**) FDH of SIS under 800 MPa and its PDF.

Table 4 also shows the distribution parameters of SIS and SII estimated by an LG. *μ*_SIS_ and *σ*_SIS_ represent the logarithmic expectation and logarithmic life standard deviation of SIS under an LG distribution, respectively, and *μ*_SII_ and *σ*_SII_ represent those of SII. In Table 4, it can be found that *μ*_SII_ is larger than *μ*_SIS_ under all three stress levels. This phenomenon is different from the conclusion reported by many investigations that the internal defects such as pores and LOFs have harmful influences on the fatigue life of an AM material [39,44]. Figure 9a,b show the FDHs of the fatigue lives of SIS under 800 MPa and SII under 720 MPa, respectively, and the PDF curves of LG_SII_ under 720 MPa and LG_SIS_ under 800 MPa are also plotted in the figures. It can be inferred that, even for a single failure behavior, SIS or SII, the LG cannot reflect the fatigue life distribution well.

**Figure 10:**
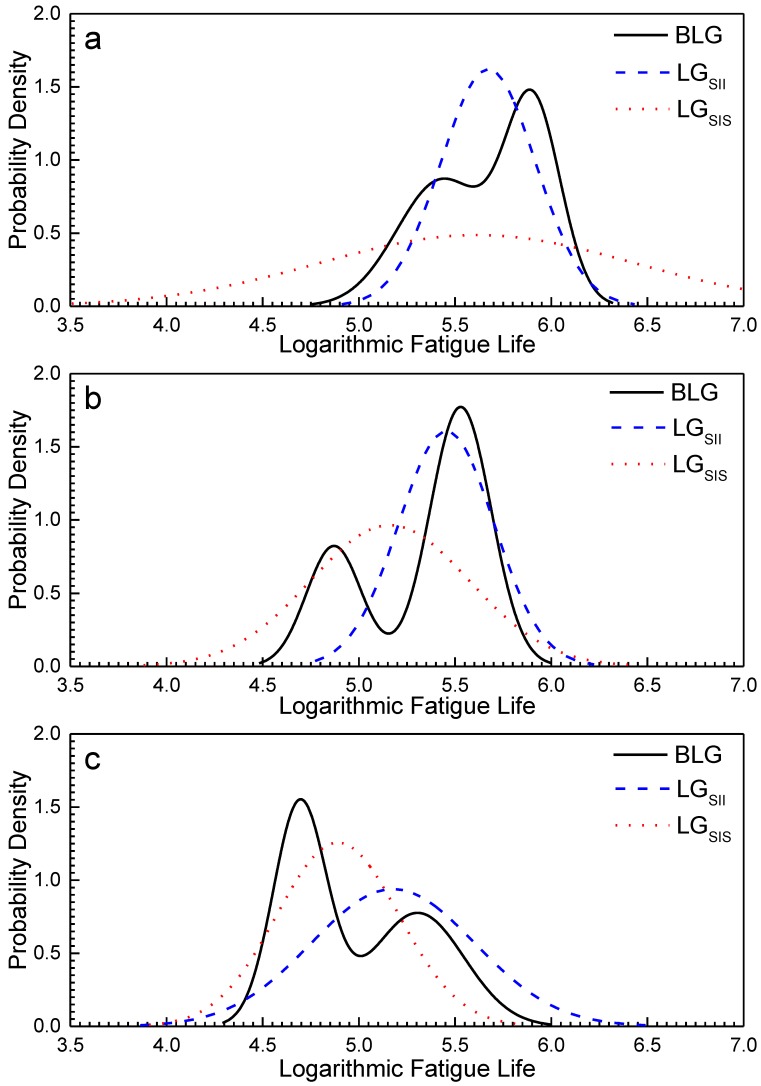
Comparisons of the PDFs of LG_SII_, LG_SIS_, and BLG under (**a**) 720, (**b**) 760, and (**c**) 800 MPa.

Figure 10 shows the PDF curves of SIS and SII under an LG and for all specimens under a BLG. The two peak positions of the BLG significantly differ from those of the LG_SII_ and LG_SIS_. Therefore, the double-peak characteristic of the LMD titanium alloy fatigue cannot be simply explained by the difference in the CIS. The difference in the CIS is one of the reasons for the large variation in the fatigue life of LMD titanium alloys and the double-peak characteristic, but it is not the only reason.

### 6.2. Influence of Size and Location of Pore Defects on the Fatigue Life

Many studies have reported the influence of the size and location of the internal defects on the fatigue life of AM specimens [13,23,24,25,26]. A positive or negative correlation between the fatigue life and the size and location of the pore defects appears to exist based on these studies. However, a different conclusion has been obtained in this paper. 

**Figure 11:**
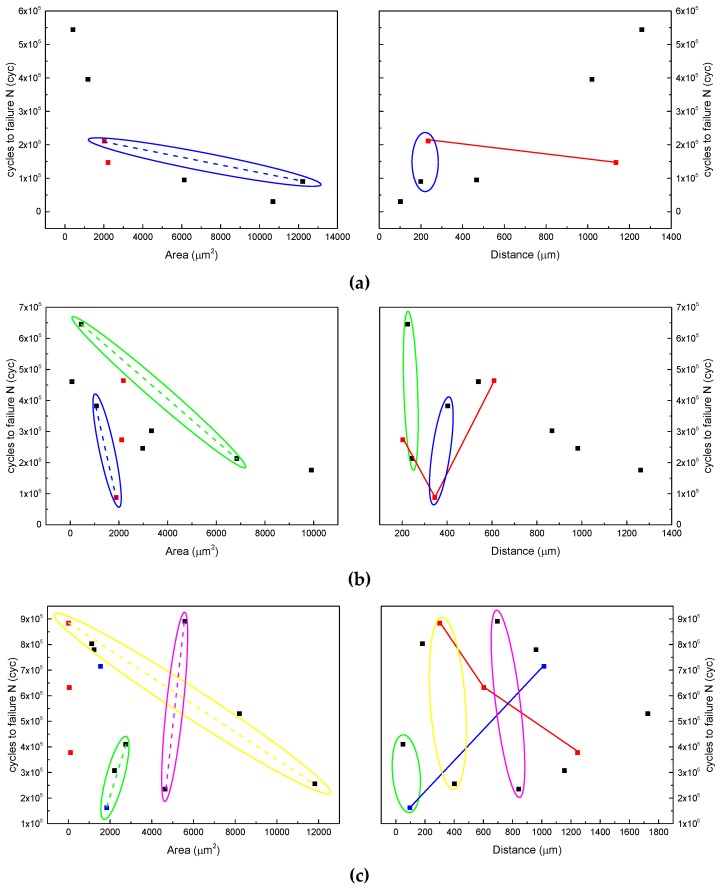
Influence of the size and location of a pore on the fatigue life under (**a**) 800, (**b**) 760, and (**c**) 720 MPa.

Figure 11 shows the influence of the size and location of a pore on the fatigue life under three stress levels. The left figures show the relationship between the fatigue life and pore area, while the right figures show the relationship between the fatigue life and the distance from the pore to the surface. Under a specific stress level, the data points that have similar areas were marked in the same color and were connected with straight lines to indicate the influence of the distance to the specimen surface in the right figures. Likewise, the data points that share a similar distance to the surface were circled and were connected with a dashed line to indicate the influence of the pore area. The previous conclusion in [26] was based on the data points under 800 MPa shown in Figure 11a. However, some of the data points under the other two stress levels showed different phenomena. In Figure 11b, the red data points show that when the areas are similar, a shorter distance does not necessarily lead to a longer fatigue life, and the blue point data in Figure 11c show a similar phenomenon. In Figure 11c, the data points circled in yellow and pink also show that, when the distances are similar, a larger area does not necessarily lead to a shorter fatigue life. Therefore, there is no significant positive or negative correlation between the fatigue life and the size and location of a pore defect. The conclusions from previous studies about the influences of the size and location of pore defects on the fatigue life [13,23,24,25,26] lack generalizability and thus need to be further investigated.

### 6.3. Comparisons of the P-S-N Curves Based on a BLG and LG

**Figure 12:**
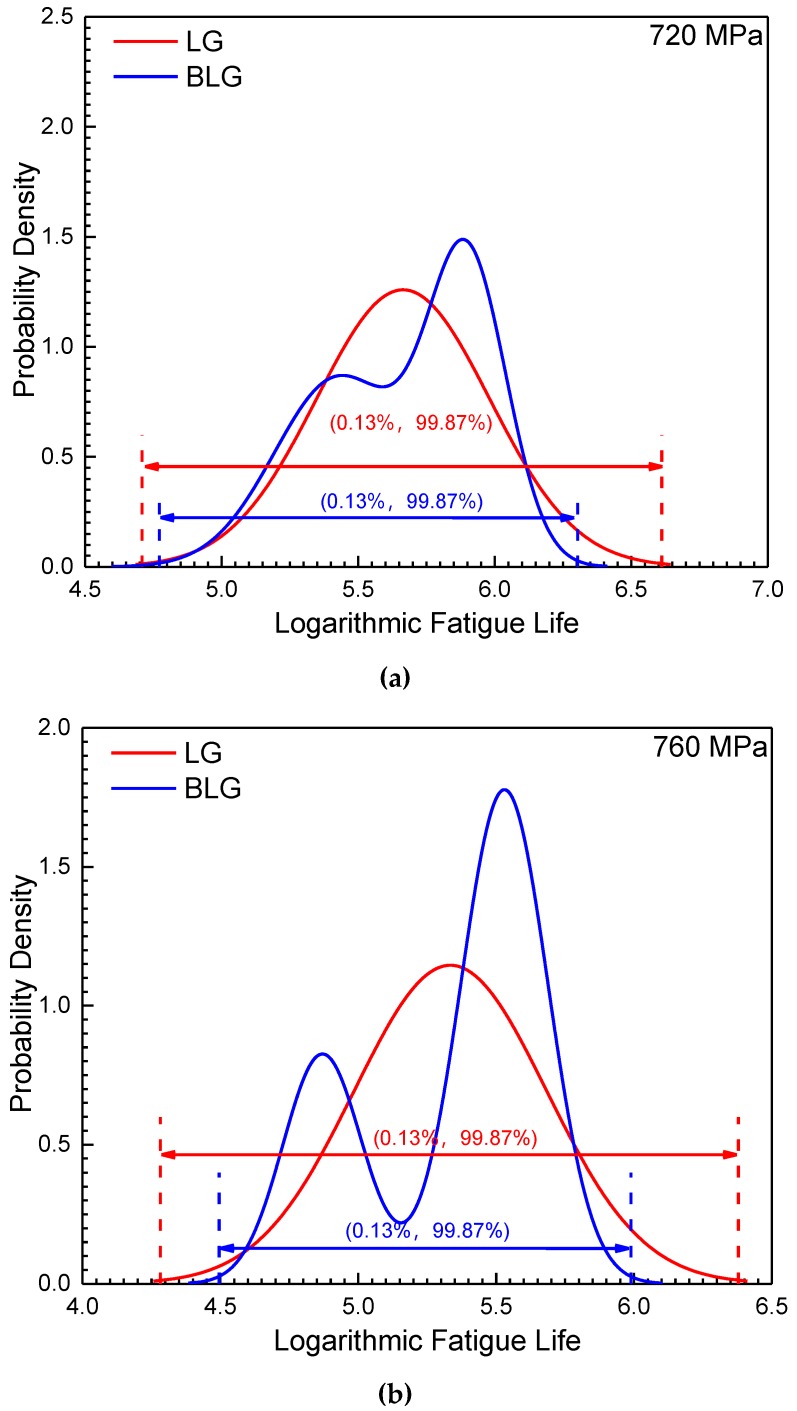

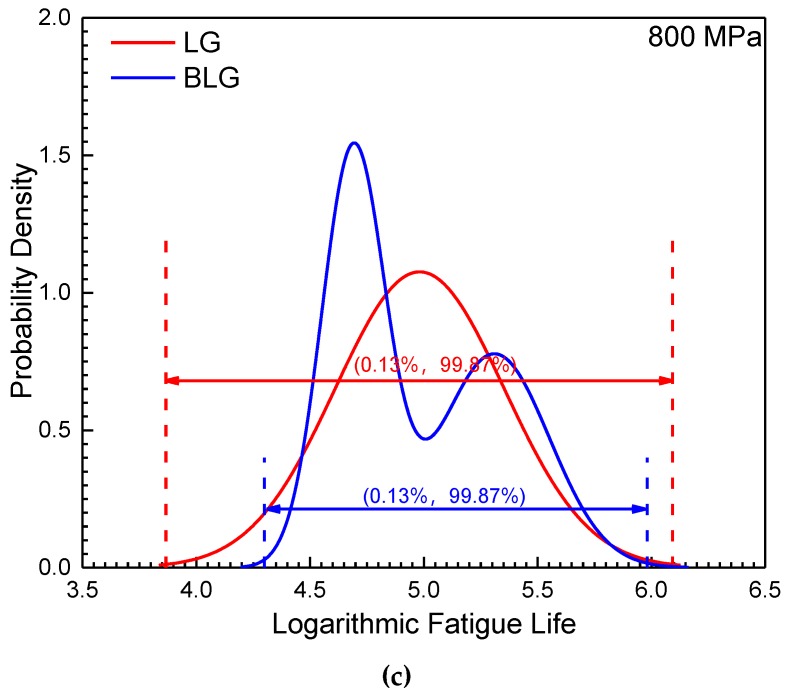
Variations in the BLG and LG. (**a**): 720; (**b**):760; (**c**):800 MPa.

The logarithmic fatigue life distribution intervals (0.13%, 99.87%) of the BLG under three different stress levels equal the intervals of ±3σ under the LG and are plotted in Figure 12. The intervals (0.13%, 99.87%) of the BLG are obviously narrower than those of the LG, indicating that the BLG model can better fit the fatigue life distribution of LMD titanium alloys without excessively amplifying their variation. Therefore, the fatigue properties of LMD titanium alloys can be more accurately described by using the *P-S-N* curve description model based on a BLG.

**Figure 13:**
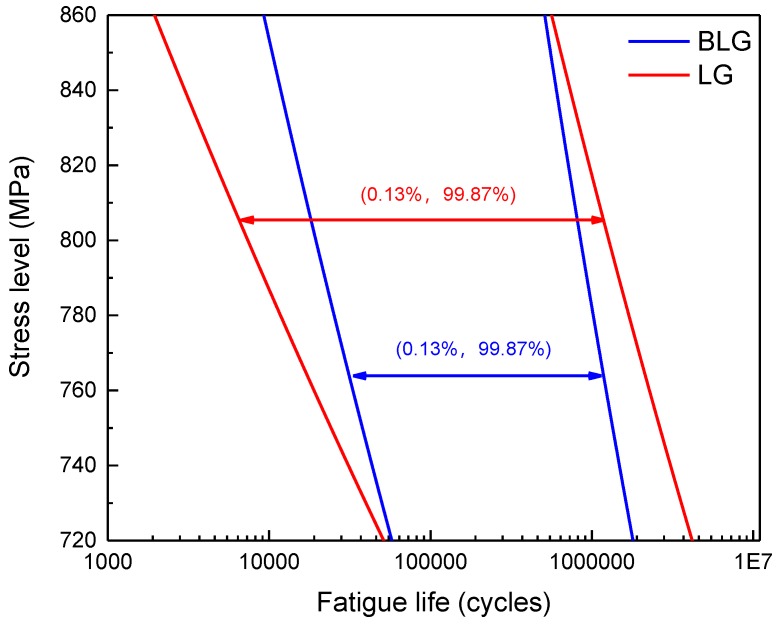
Variation of P-S-N curves based on an LG and BLG.

The *P-S-N* curve based on an LG was estimated by using a method similar to that in Section 5. Figure 13 shows the (0.13%, 99.87%) distribution bands of the *P-S-N* curves based on both an LG and BLG. The distribution band of the *P-S-N* curves based on the BLG is significantly narrower than that of the *P-S-N* curves based on the LG.

**Figure 14:**
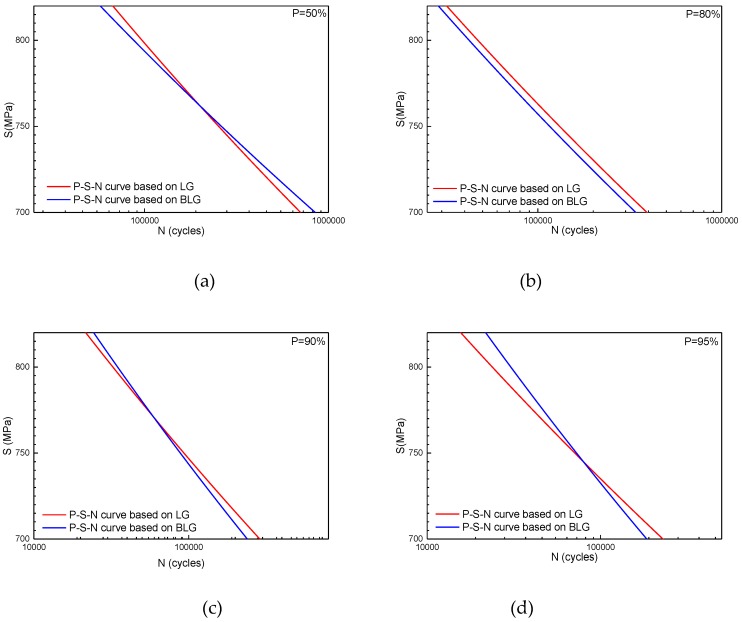

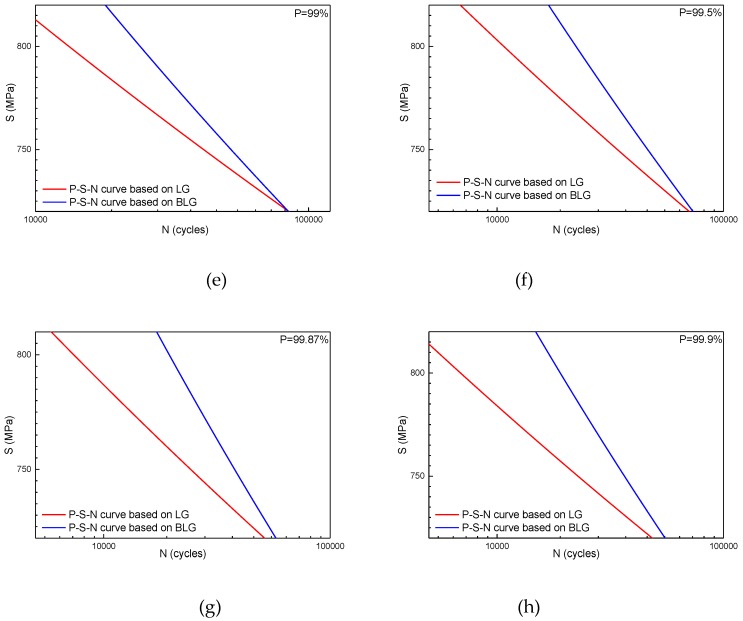
*P-S-N* curves based on an LG and BLG under reliabilities of (**a**) 50%, (**b**) 80%, (**c**) 90%, (**d**) 95%, (**e**) 99%, (**f**) 99.5%, (**g**) 99.87%, and (**h**) 99.9%.

Table 5 gives the estimated parameters of the *P-S-N* curves based on an LG under several reliability values. The parameters show different laws compared with those of the *P-S-N* curves based on a BLG, as shown in Table 3. Both *C_P_* and *m_P_* increase with the reliability value, *P*. *P-S-N* curves based on an LG and BLG are plotted in Figure 14. Table 6 shows the fatigue life at a typical reliability value under three stress levels, 720, 760, and 800 MPa, which was obtained from the two types of *P-S-N* curves. The figures and tables above indicate that the *P-S-N* curve model based on a BLG can not only more accurately reflect the fatigue life properties but also significantly increase the fatigue life at high-reliability values. The fatigue life at a reliability value of 99.9% is improved by almost two-fold in the high stress level regime (800 MPa). Therefore, describing the fatigue properties of LMD titanium alloys by using the *P-S-N* model based on a BLG can greatly promote the applications of LMD titanium alloys in engineering, especially in load-bearing structures, significantly improve the structural design flexibility, and reduce the structure weight.

## 7. Conclusions and Recommendations for Further Work

In this paper, fatigue tests of standard smooth Ti-6.5Al-2Zr-1Mo-1V specimens were conducted, and abundant data were obtained. The mixed failure behavior and the influence of internal pores on the fatigue life of LMD Ti-6.5Al-2Zr-1Mo-1V specimens were discussed in detail, and a novel *P-S-N* curve method based on a BLG was established to describe the fatigue properties of LMD titanium alloys. Through the method, the fatigue life at a high reliability value is increased. This result may promote the application of LMD titanium alloys in engineering and can significantly improve the structural design flexibility and reduce the structure weight. From the test results and analyses, the following conclusions can be drawn:

(i) Mixed failure behaviors of Ti-6.5Al-2Zr-1Mo-1V titanium alloy, with cracks initiating from both internal pores and the surface or subsurface, are observed in the medium- and high-cycle fatigue regimes. These mixed failure behaviors may be an intrinsic characteristic of LMD titanium alloys under CA stress and independent of the stress level. However, the proportion of SII in the overall population may be related to the stress level: the proportion decreases with an increase in the stress level.

(ii) For the internal pore failure model, there is no significant positive or negative correlation between the fatigue life and the size and location of the pore defects. 

(iii) The BLG is reasonable for the fatigue life description in the medium- and high-cycle fatigue regimes of LMD titanium alloys. The Newton–Raphson algorithm was used to estimate the BLG parameters based on the MLE method, and the Basquin equation can be used to describe the *P-S-N* curve of the LMD titanium alloy. 

There are also some recommendations for further investigations:

(i) In-depth research on the relationship between the proportion of SII and the stress level is needed. This phenomenon may be caused by the combined influences of the microstructure, the types and geometric parameters of the defects, and the local stress state.

(ii) The influence of the sizes and locations of the pore defects on the fatigue life requires further research to arrive at a more general conclusion.

## Figures and Tables

**Table 1:** Fatigue life distribution under a lognormal distribution (LG).

Stress Level/MPa	Number of Specimens	*μ*_LG_	*σ*_LG_	*y*_max_/Cycles	*y*_min_/Cycles	$N_{50,LG}/\mathrm{Cycles}$
720	15	5.66	0.316	108,089	1,559,872	457,088
760	17	5.33	0.348	49,758	645,171	213,796
800	22	4.98	0.370	30,715	544,102	95,499

**Table 2:** Bimodal lognormal distribution (BLG) parameters estimated by maximum likelihood estimation (MLE).

Stress Level/MPa	*α*	*μ*_1_	*σ*_1_	*μ*_2_	*σ*_2_
720	0.508	5.43	0.235	5.90	0.144
760	0.296	4.87	0.143	5.53	0.158
800	0.528	4.69	0.138	5.31	0.242

**Table 3:** Parameters of the *P-S-N* curve based on a BLG.

*P*	*m_P_*	*C_P_*	*r*
50%	17.01	2.07 × 10^54^	0.965
80%	15.62	9.51 × 10^49^	0.996
90%	14.45	3.08 × 10^46^	0.991
95%	13.52	5.56 × 10^43^	0.990
99%	11.88	7.33 × 10^38^	0.992
99.5%	11.29	1.37 × 10^37^	0.994
99.87%	10.29	1.49 × 10^34^	0.996
99.9%	10.11	4.39 × 10^33^	0.997

**Table 4:** LG parameters of SIS and SII under three stress levels.

*S*/MPa	*n*	*n*_SIS_	*μ*_SIS_	*σ*_SIS_	*n*_SII_	*μ*_SII_	*σ*_SII_	*n*_SII_/*n*
720	15	2	5.61	0.819	13	5.67	0.246	0.867
760	17	7	5.16	0.414	10	5.46	0.248	0.588
800	22	15	4.89	0.317	7	5.18	0.424	0.318

**Table 5:** Parameters of the P-S-N curve based on an LG.

*P*	*m_P_*	*C_P_*		*r*
50%	14.85	1.28 × 10^48^	0.999	
80%	15.85	4.82 × 10^50^		1.000
90%	16.36	1.07 × 10^52^		1.000
95%	16.98	4.65 × 10^53^		1.000
99%	17.60	1.68 × 10^55^		1.000
99.5%	17.90	9.74 × 10^55^		1.000
99.87%	18.41	2.09 × 10^57^		1.000
99.9%	18.51	3.64 × 10^57^		1.000

**Table 6:** Fatigue life calculated from P-S-N curves under specific reliabilities (cycles).

*P*	720 MPa		760 MPa		800 MPa	
	LG	BLG	LG	BLG	LG	BLG
50%	463,447	523,842	207,587	20,8930	96,917	87,337
80%	250,726	219,381	106,365	94,276	47,206	42,306
90%	181,635	159,221	75,024	72,912	32,389	34,738
95%	142,167	126,241	56,755	60,758	23,747	30,367
99%	84,723	84,489	32,689	44,422	13,256	24,165
99.5%	70,599	73,384	26,816	39,847	10,705	22,325
99.87%	51,095	59,168	19,143	31,282	7,341	20,030
99.9%	48,440	55,208	17,816	31,945	6,893	19,020
